# Maternal stress during the third trimester of pregnancy and the neonatal microbiome

**DOI:** 10.1080/14767058.2023.2214835

**Published:** 2023-12

**Authors:** Sandra J. Weiss, Maryam Hamidi

**Affiliations:** a Department of Community Health Systems, University of California, San Francisco, San Francisco, CA, USA; b School of Health and Natural Sciences, Dominican University of California, San Rafael, CA, USA

**Keywords:** Microbiome, maternal stress, neonate, pregnancy, fetal exposure

## Abstract

**Objectives:**

Preliminary research suggests that maternal prenatal stress may alter the development of the fetal microbiome and resulting microbial composition after birth. However, the findings of existing studies are mixed and inconclusive. The purpose of this exploratory study was to assess whether maternal stress during pregnancy is associated with the overall number and diversity of various microbial species in the infant gut microbiome or the abundance of specific bacterial taxa.

**Methods:**

Fifty-one women were recruited during their third trimester of pregnancy. The women completed a demographic questionnaire and Cohen’s Perceived Stress Scale at recruitment. A stool sample was collected from their neonate at one month of age. Data on potential confounders, such as gestational age and mode of delivery, were extracted from medical records to control for their effects. 16s rRNA gene sequencing was used to identify the diversity and abundance of microbial species, along with multiple linear regression models to examine the effects of prenatal stress on microbial diversity. We employed negative binomial generalized linear models to test for differential expression of various microbial taxa among infants exposed to prenatal stress and those not exposed to prenatal stress.

**Results:**

More severe symptoms of prenatal stress were associated with a greater diversity of microbial species in the gut microbiome of neonates (β = .30, *p* = .025). Certain microbial taxa, such as *Lactobacillus* and *Bifidobacterium*, were enriched among infants exposed to greater maternal stress in utero, while others, such as *Bacteroides* and *Enterobacteriaceae*, were depleted in contrast to infants exposed to less stress.

**Conclusions:**

Findings suggest that mild to moderate stress exposure in utero could be associated with a microbial environment in early life that is more optimally prepared to thrive in a stressful postnatal environment. Adaptation of gut microbiota under conditions of stress may involve upregulation of bacterial species, including certain protective microorganisms (e.g. *Bifidobacterium*), as well as downregulation of potential pathogens (e.g. *Bacteroides*) *via* epigenetic or other processes within the fetal/neonatal gut-brain axis. However, further research is needed to understand the trajectory of microbial diversity and composition as infant development proceeds and the ways in which both the structure and function of the neonatal microbiome may mediate the relationship between prenatal stress and health outcomes over time. These studies may eventually yield microbial markers and gene pathways that are biosignatures of risk or resilience and inform targets for probiotics or other therapies in utero or during the postnatal period.

## Introduction

The time from conception through the second year of life is a crucial stage of development, during which foundations for later health are established and are influenced substantially by environmental conditions [1]. Therefore, prenatal maternal stress and its effects on the uterine environment may have important developmental implications. Intrauterine exposure to maternal stress has been associated with adverse outcomes such as preterm birth and a greater risk of childhood behavioral and physical health problems later in life [2–4]. Potential mechanisms underlying the effects of prenatal stress are not yet clear; however, one hypothesized mechanism involves the alteration of the fetal gut microbiome [3,4]. To date, ourknowledge of the relationship between prenatal stress and the microbiome of newborns is limited [1,3,5].

Only two studies have examined the association between maternal stress during pregnancy and the overall number and balance of various bacteria (i.e. diversity) in the infant gut microbiome. In examining the diversity of microbial taxa in the infant mouse microbiome, Gur et al. [6] found no differences between offspring exposed to prenatal stressors and those not exposed. Similarly, Aatsinki et al. [7] reported no relationship between prenatal stress and the overall diversity of neonatal microbial taxa in human infants.

However, both animal (*N* = 3) and human (*N* = 2) studies have noted differences in the relative abundances of specific bacterial taxa and microbial species. Two animal and one human studies reported that prenatal stress was associated with depleted *Lactobacillus* in the neonatal gut [8–10]. Two studies (one animal and one human) also found that prenatal stress is related to reduced *Bifidobacteria* [8,10]. In addition, two human studies have reported that increased *Proteobacteria* was associated with pregnancy stress [7,10]. Other findings have been reported in only one study each, including decreased *Bacteroides* [6] and *Akkemansia* [7], and increased *Anaerotruncus, Peptococcus* and *Oscillibacter* [9]. A final study with human infants found no differences in microbial diversity or abundance of specific taxa between infants whose mothers reported prenatal perceived stress and those who did not [1].

Although a few results have been supported across these preliminary studies (i.e. effects on *Lactobacillus, Bifidobacteria*, and *Proteobacteria*), the findings are mixed overall. In addition, only three studies have been conducted on human mothers and infants. This exploratory study sought to address these gaps. Our aim was to assess the relationship between maternal stress during pregnancy and the diversity and composition of the neonate gut microbiome at one month of age. We explored whether prenatal stress is associated with the richness or evenness of different species represented in the infant gut microbiome and the degree to which exposure to greater prenatal stress is related to the differential abundance of specific microbial taxa in the infant gut microbiome.

## Material and methods

### Recruitment and study design

Women were recruited during their third trimester of pregnancy from two obstetric clinics affiliated with a large university medical center. The inclusion criteria were being 18 years or older and speaking English or Spanish. Women were excluded if they had ongoing steroid use or a history of endocrine conditions, smoked, serious medical problems, or cognitive impairment. Infants were excluded if they had chromosomal or genetic anomalies, chronic lung disease, congenital heart disease, or other major neonatal illnesses. Dyads were also excluded from the analysis if either the woman or infant received antibiotics during the study period. Eligible women were provided with information about the study, and their consent was acquired by a member of the research team. The Institutional Review Board of the University of California, San Francisco approved the research and informed consent process (IRB# 14-13516).

The women completed questionnaires on their demographics and perceived stress at enrollment. Stool samples were acquired from infants at one month of age for analysis of the gut microbiome. Information on potential covariates, including data on their treatment with antenatal corticosteroids, obstetric risk, mode of delivery (vaginal vs cesarean), gestational age, neonatal morbidity and method of feeding their infant (breast fed or formula), was also acquired.

### Measures

#### Perceived stress

Cohen’s Perceived Stress Scale (PSS) was used to measure pregnancy stress [11]. The PSS assessed the degree to which women felt that their lives were unpredictable, uncontrollable, and overloaded with stressors over the prior four-week period. The scale has 10 items, each rated on a five-point Likert scale. The total stress score was developed from the sum of all items. The PSS has shown excellent construct, discriminant, and predictive validity, as well as test-retest reliability across cultures and languages [11–13].

#### Microbial composition

DNA/RNA Shield fecal collection tubes (Zymo Research) were used to collect stool specimens. A stool sample consisting of the infant’s first bowel movement of the day was gathered from the infant’s diaper by the mother. The samples were stored in a freezer until they were transported on ice to the microbiome lab where they were processed.

Purification and PCR amplification of the V4 hypervariable region of the 16S rRNA gene were performed after isolation of the DNA from the sample, followed by DNA sequencing on an Illumina NextSeq. A 97% sequence similarity threshold was used to bin aligned read pairs containing less than two expected errors into operational taxonomic units (OTUs), with OTUs of non-bacterial origin or common contaminants discarded. The maximum read count of each remaining OTU in any single extraction control was subtracted from the read count of that OTU for each sample.

Two different diversity indices were used as dependent variables: (1) the Simpson Index, which, although measuring both richness and evenness, has a primary emphasis on richness (number of different taxa present), and (2) the Shannon Index, which has a primary emphasis on evenness (extent to which taxa are in even abundance with one another), although this index also assessed both richness and evenness [14,15].

#### Covariates

Previous research suggests the potential relationship of exposure to antenatal corticosteroids, obstetric risk, mode of delivery, gestational age, neonatal morbidity, and method of feeding to microbiome composition [16]. Thus, information was acquired from the electronic medical record and a maternal questionnaire to control for their potential effects. We used the *Obstetric Risk Index* to calculate risk data from medical records, with higher scores indicating more medical complications and problems during pregnancy [17]. Neonatal morbidity was determined using the *Morbidity Assessment Index for Newborns*, which denotes the severity of an infant’s health problems during the first week of life [18].

#### Data analysis

Preliminary correlations and analyses of variance were computed to determine bivariate relationships between covariates and measures of microbial diversity (Shannon and Simpson indices). Two separate multiple linear regressions were then employed to examine the relationship between pregnancy stress and the Shannon and Simpson indices. Covariates showing significant associations in the preliminary testing were included in all subsequent diversity and abundance tests.

Differential gene expression analysis was performed to compare the composition of various microbial taxa between infants whose mothers experienced higher and lower pregnancy stress. We used Bioconductor’s DESeq2 package [19] to detect differentially expressed genes in the two groups from the OTU count data. Raw count data were normalized by accounting for the effect size prior to differential abundance calculations based on the negative binomial Wald test. The results were adjusted for multiple testing with the Benjamini–Hochberg procedure, which controls for the false discovery rate. NCBI’s RefSeq was the reference database used for taxonomic classification [20].

## Results

Data from 51 women and their infants were analyzed. Fifty-three percent of the dyads were racially diverse, including 28% Asian Americans (*n* = 14), 19% Native American/Pacific Islander (*n* = 10), and 6% African American/black (*n* = 3). The remaining 47% (*n* = 24) were European-American/white. Thirty-two percent (*n* = 16) were Hispanic/Latina. The age of the women ranged from 21 to 43 years, with a mean of 33.6 (SD = 5.38). Sixty percent (*n* = 30) of the women had a vaginal delivery in contrast to a cesarean birth, 64% (*n* = 34) breast fed their infants, and 69% (*n* = 35) received antenatal corticosteroids as part of obstetric care. On average, their stress level was on the cusp of low to moderate stress (*M* = 13.23, SD = 6.39), with a score of 14 indicating moderate stress. 57% (*n* = 29) of women fell into the low stress category, while 43% were in the moderate to high stress category (*n* = 22). They had a mean of three obstetric risks, with a range of 0 to 7. The infants ranged from 31 to 41 weeks of gestation (*M* = 35.28, SD = 2.17), and 51% were females (*n* = 26). The infant scores for neonatal morbidity ranged from 0 (no medical problems) to 805. The mean morbidity was 165 (SD = 187) out of a possible score >2000.

### Preliminary assessment of covariates

Only two of the six covariates showed significant relationships with Shannon and Simpson microbial diversity measures in preliminary analysis. Gestational age (GA) was positively associated with both Shannon (*r* = .33) and Simpson (*r* = .37) indices at *p*<.01. Neonatal morbidity was negatively related to both Shannon (*r* = −.29) and Simpson (*r* = −.29) indices at *p*<.05. Thus, we adjusted for GA and neonatal morbidity in all subsequent analyses. Antenatal corticosteroids, obstetric risk, mode of delivery, and method of feeding showed no associations with microbial composition in bivariate analyses.

### The association of perceived stress to microbial diversity

The results of the linear regression analysis are shown in Table 1. After controlling for the effects of gestational age and neonatal morbidity, prenatal stress was significantly associated with the diversity of the infant gut microbiome when measured using the Simpson Diversity Index (β = .30, *p* = .02). Infants exposed to more severe symptoms of maternal stress *in utero* had a greater number of different taxa in their gut microbiomes. Although the relationship between prenatal stress and microbial diversity showed a trend, it did not reach significance for the Shannon Diversity Index (β = .25, *p* = .06). Neither covariate was significantly associated with microbial diversity.

### The association of perceived stress to microbial composition

**Figure 1.**
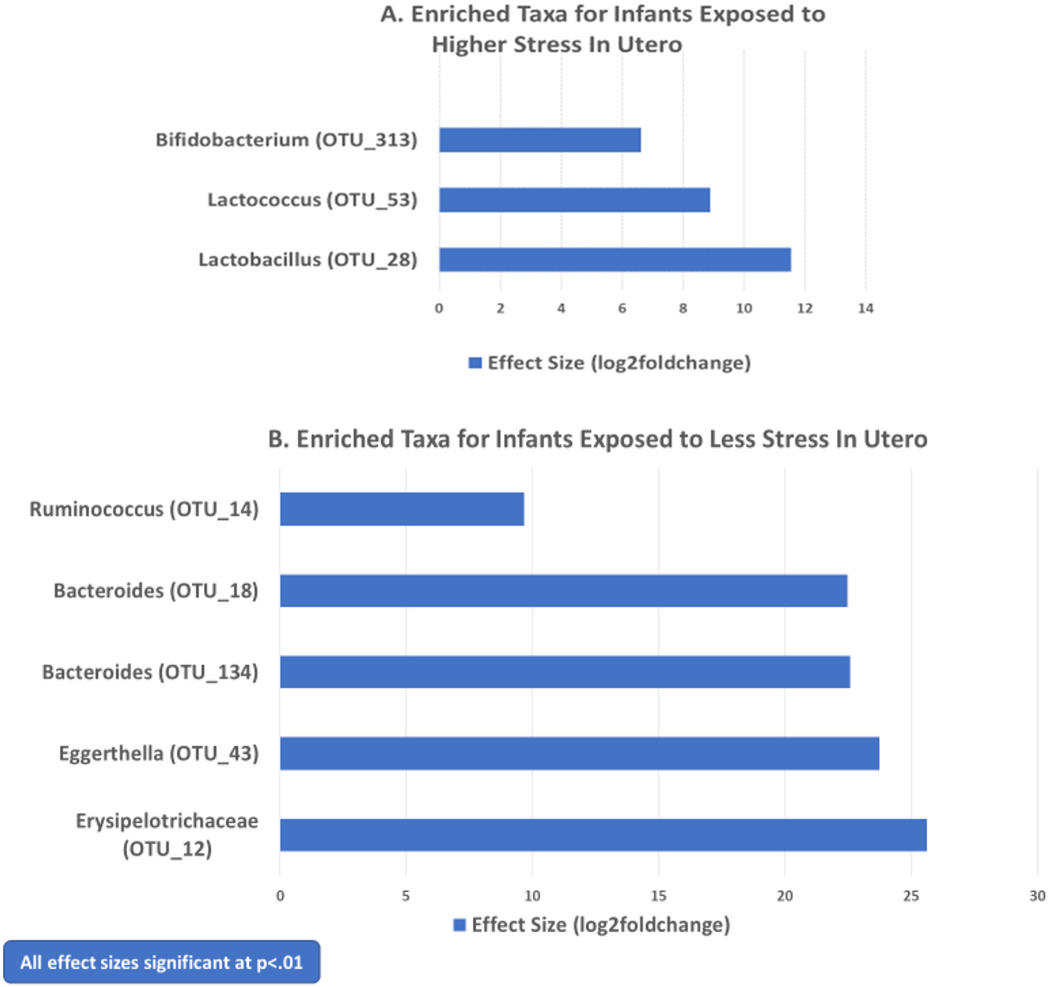
Bacterial taxa showing significant differences in microbial abundance for neonates exposed to higher versus lower maternal stress in utero.

Figure 1 displays bacterial taxa that were significantly different in the gut microbiome of infants whose mothers experienced higher levels of stress during pregnancy versus those whose mothers were less stressed. Figure 1(A) shows log2foldchange for the three OTU’s that showed significantly greater abundance (more enriched) in the samples of infants’ exposed to more stress: *Lactobacillus* (OTU_28; 11.55, *p*<.001)*, Lactococcus* (OTU_53; 8.88*, p*<.01) and *Bifidobacterium* (OTU_313; 6.62, *p*<.01). Figure 1(B) presents log2foldchange values for the 5 OTU’s that were significantly higher in abundance (*p*<.01) for the infants whose mothers were less stressed during pregnancy than those experiencing more stress. These included *Erysipelotrichaceae* (OTU_12; 25.62*, p*<.001), *Eggerthella* (OTU_43; 23.74, *p*<.001)*, Bacteroides* (OTU_134; 22.57, *p*<.001)*, Bacteroides* (OTU_18; 22.46, *p*<.001), and *Ruminococcus* (OTU_14; 9.66, *p*<.01). *Enterobacteriaceae* (OTU_298; 2.63*, p*<.05) and *Enterococcus* (OTU_262; 3.66, *p*<.05) were also more enriched in the gut microbiome of infants experiencing less stress exposure *in utero*, although the *p* values of these taxa were not as small.

## Discussion

The results indicate that fetal exposure to maternal stress is associated with greater diversity in the number of different bacterial taxa in the gut microbiome of infants at one month of age. In addition, there were significant differences in the abundance of certain microorganisms between infants exposed to greater versus lesser prenatal stress.

### Diversity of the microbiome

Our finding that prenatal stress was associated with greater microbial diversity differs from two previous studies that found no effect of prenatal stress on the number and diversity of infant gut microbes [6,7]. However, one study used a mouse model that examined exposure to stressors rather than *perceived stress* of the mother. Another study grouped women into categories of psychological distress based on measures that were not all stress-specific and combined responses across three time points of pregnancy (we focused on the third trimester). They also analyzed infant microbiome data at 2.5 months postnatal rather than at 1 month, as in our research. Our specific assessment of perceived stress and the closer time congruence between our pregnancy and microbiome measurements may have enabled a more targeted evaluation of the relationship between prenatal stress and *neonatal* microbial diversity.

In general, greater gut microbial diversity has been viewed as a beneficial state associated with better health status and resistance to environmental stressors [21–23]. Thus, our finding that exposure to stress is associated with greater diversity was unexpected. However, other studies have provided evidence of greater diversity in what is considered a less optimal context. For instance, formula-fed infants have more diverse microbiomes than breastfed infants during the first months of life [24–26]. It has been proposed that complex, more diverse microbial communities are inherently vulnerable to destabilization and may not necessarily be optimal under all conditions [27], including in the early months of development [25]. Research needs to examine the trajectory of microbial diversity as infant development proceeds and the ways in which a more diverse microbial environment in the first month of life may mediate the relationship between prenatal stress and health outcomes over time.

### Differential abundance of specific microorganisms

In addition, we found that infants exposed to more stress had significantly higher levels of bacteria typically viewed as beneficial, including *Lactobacillus* and *Bifidobacterium.* In contrast, the microbiomes of infants exposed to less stress were more abundant in bacterial taxa such as *Bacteroides, Eggerthella*, and *Enterobacteriaceae.* These bacteria are commonly associated with health compromising effects, such as neonatal sepsis and necrotizing enterocolitis [28,29].

### Species-level effects

Although taxa such as *Lactobacillus* and *Bifidobacterium* have been viewed as beneficial, specific strains of these bacteria have been implicated in serious clinical infections [30], including in infants [31]. Future research must examine the links between prenatal stress and targeted microbial species to better understand the potential pathogenic effects of stress.

### Adaptive mechanisms

Microbial adaptive mechanisms in response to stress may enhance the enrichment of bacteria that are traditionally considered beneficial. Gut microbiota are capable of resisting perturbation from stress, mitigating its negative effects, and recovering a healthy state following exposure to stress to repair damage and restore growth or survival potential [32–34]. Some adaptive mechanisms involve coordinated regulation systems that change the pattern of gene expression in the cell and cellular processes [35]. This might occur through upregulation of certain protective microorganisms (e.g. *Bifidobacterium*) or downregulation of potential pathogens (e.g. *Bacteroides*) *via* epigenetic or other processes within the gut-brain axis [36]. Such adaptive capacities are ubiquitous in diverse microbial environments [37], such as those we found among infants exposed to prenatal stress.

### Beneficial effects of stress

Research indicates that stressors can affect gut microbiota in positive ways [32], especially mild to moderate stress exposure, such as that incurred by infants in our study [38]. Thus, prenatal stress may catalyze or enhance the growth of probiotics, such as *Lactobacillus* and *Bifidobacterium.* In line with this view, Kirby et al. [39] demonstrated that mild to moderate stress during pregnancy has numerous beneficial effects on offspring, including stem cell growth and neuronal generation. Prenatal stress may program the development of a microbiome *in utero* that is biologically better prepared (i.e. with an abundance of health-promoting versus health-compromising bacteria) to thrive in a potentially stressful environment.

### Limitations

Although the participants were racially and ethnically diverse, the sample size was modest. We acquired one stress assessment during the third trimester; however, the effects of stress in other trimesters are unknown. We were not able to characterize microbiota at the species level. In addition, we did not examine associations between prenatal stress and the microbiome immediately at birth or in early postnatal weeks and do not know whether the effects of stress differ as development proceeds beyond one month of age. Lastly, we did not examine the role of the maternal gut microbiome, which we are currently assessing in further research.

## Conclusions

This study provides novel results on the potential effects of prenatal stress on neonatal microbiome development. These findings that mild-to-moderate stress exposure *in utero* could be associated with a microbial environment in early life that is more optimally prepared to thrive in a stressful postnatal environment. However, further research is needed to understand the trajectory of microbial diversity and composition as infant development proceeds and the ways in which both the structure and function of the neonatal microbiome may mediate relationships between maternal symptoms of prenatal stress and health outcomes over time. In addition, research must examine the links between prenatal stress and different microbial strains, their nuanced functions, and the underlying epigenetic mechanisms that influence microbial expression to fully understand the harmful or beneficial effects of stress. Studies that build on our findings may eventually yield microbial markers and microbial gene pathways that are biosignatures of risk or resilience and inform targets for probiotic or other therapies *in utero* or during the postnatal period.

## Figures and Tables

**Table 1.** Regression model for the relationship of maternal prenatal stress to diversity of the neonatal microbiome, adjusting for gestational age and neonatal morbidity.

	Beta	SE	*p*	95% confidence intervals
Shannon Diversity Index*				
Neonatal gestational age	.18	.04	.26	−.034, .140
Neonatal morbidity	−.21	.00	.18	−.002, .000
Maternal stress	.25	.01	.06	−.002, .054
Simpson Diversity Index**				
Neonatal gestational age	.21	.01	.16	−.010, .056
Neonatal morbidity	−.20	.00	.19	−.001, .000
Maternal stress	.30	.01	.02	.002, .022

* Model F = 3.65, *p* = .019;

** Model F = 4.87, *p* = .005.

## Data Availability

The data that support the findings of this study are available from the corresponding author [SJW] upon reasonable request.
